# Relation of Exercise to Health

**Published:** 1887-07

**Authors:** 


### RELATION OF EXERCISE TO HEALTH.

The relation of exercise to health is plain. Any part that is not used gradually wastes and atrophies. The fishes in Mammoth cave have no use for eyes in that darkness, and nature shows her disapproval of sinecure positions by taking away their organs of sight. But similarly, the overuse of a part attracts to that part more than its share of nutriment, and congestion or hyperethesia results. If then, the nerves are oversensitive, the circulation should be diverted into the muscles. Disease seldom finds a lodgment when there is a free and unimpeded circulation of the blood to every part of the body.

Nearly the sum total of exercise taken by those not engaged in manual labor is restricted to the lower part of the body, while the upper two-thirds, which contains the vital organs, is almost wholly neglected. Thus it is that we are flat-chested and subject to pulmonary difficulties, and for want of exercise the muscles which support the internal organs are ill-nourished and flaccid, and yield to any sudden strain, or sometimes, spontaneously, it would seem, collapse. Given a well-conditioned body, a very little exercise, if taken regularly and systematically, will keep it so, but without the expenditure of physical strength good health cannot be maintained.

Systems of exercise are often criticised as artificial. It is, of course, of no more value to any one that he should be able to swing clubs or to go through a dumb-bell drill correctly for its own sake than that a business man should be able to repeat Newton's Binomial Theorem or demonstrate on demand a proposition of Euclid ; but the discipline previously gone through is mental capital on the one hand and physical capital on the other. If a scheme of exercise was ever needed to bring into harmonious relations the different parts of the body and to maintain the equilibrium between them, it is needed here in America, the center of nervous life and activity. Such a system is attainable and practicable.—*Good Housekeeping*.

